# Consensus statement from the International Consensus Meeting on the Role of Decompressive Craniectomy in the Management of Traumatic Brain Injury: Consensus statement

**DOI:** 10.1007/s00701-019-03936-y

**Published:** 2019-05-28

**Authors:** Peter J. Hutchinson, Angelos G. Kolias, Tamara Tajsic, Amos Adeleye, Abenezer Tirsit Aklilu, Tedy Apriawan, Abdul Hafid Bajamal, Ernest J. Barthélemy, B. Indira Devi, Dhananjaya Bhat, Diederik Bulters, Randall Chesnut, Giuseppe Citerio, D. Jamie Cooper, Marek Czosnyka, Idara Edem, Nasser M.F. El-Ghandour, Anthony Figaji, Kostas N. Fountas, Clare Gallagher, Gregory W.J. Hawryluk, Corrado Iaccarino, Mathew Joseph, Tariq Khan, Tsegazeab Laeke, Oleg Levchenko, Baiyun Liu, Weiming Liu, Andrew Maas, Geoffrey T. Manley, Paul Manson, Anna T. Mazzeo, David K. Menon, Daniel B. Michael, Susanne Muehlschlegel, David O. Okonkwo, Kee B. Park, Jeffrey V. Rosenfeld, Gail Rosseau, Andres M. Rubiano, Hamisi K. Shabani, Nino Stocchetti, Shelly D. Timmons, Ivan Timofeev, Chris Uff, Jamie S. Ullman, Alex Valadka, Vicknes Waran, Adam Wells, Mark H. Wilson, Franco Servadei

**Affiliations:** 1 Division of Neurosurgery, Department of Clinical Neurosciences, Addenbrooke’s Hospital and University of Cambridge, Cambridge Biomedical Campus, Cambridge, CB20QQ UK; 2 NIHR Global Health Research Group on Neurotrauma, University of Cambridge, Cambridge, UK; 3 Division of Neurological Surgery, Department of Surgery, College of Medicine, University of Ibadan, Ibadan, Nigeria; 4 Department of Neurological Surgery, University College Hospital, Ibadan, Nigeria; 5 Neurosurgical Unit, College of Health Sciences, Addis Ababa University, Addis Ababa, Ethiopia; 6 Department of Clinical Medicine, Faculty of Medicine, University of Bergen, Bergen, Norway; 7 Department of Neurosurgery, Faculty of Medicine, Universitas Airlangga, Soetomo General Hospital, Surabaya, Indonesia; 8 Program in Global Surgery and Social Change, Department of Global Health and Social Medicine, Harvard Medical School, Boston, MA USA; 9 Department of Neurosurgery, National Institute for Mental Health and Neurosciences, Bangalore, India; 10 Wessex Neurological Centre, University Hospital Southampton, Southampton, UK; 11 Harborview Medical Center, University of Washington, Seattle, WA USA; 12 School of Medicine and Surgery, University of Milan-Bicocca, Milan, Italy; 13 Neuro-Intensive Care, Department of Emergency and Intensive Care, ASST, San Gerardo Hospital, Monza, Italy; 14 Australian and New Zealand Intensive Care Research Centre, Monash University, Melbourne, Victoria Australia; 15 Department of Intensive Care, Alfred Hospital, Melbourne, Victoria Australia; 16 Division of Neurosurgery, Department of Surgery, The Ottawa Hospital, Ottawa, ON Canada; 17 Department of Neurosurgery, Faculty of Medicine, Cairo University, Cairo, Egypt; 18 Division of Neurosurgery and Neuroscience Institute, University of Cape Town, Cape Town, South Africa; 19 Department of Neurosurgery, University Hospital of Larissa and University of Thessaly, Larissa, Greece; 20 Division of Neurosurgery, Department of Clinical Neurosciences, University of Calgary, Calgary, Alberta Canada; 21 Department of Neurosurgery, University of Manitoba, Winnipeg, Manitoba Canada; 22 Department of Neurosurgery, Azienda Ospedaliero Universitaria di Parma, Parma, Italy; 23 Department of Neurosurgery, Christian Medical College, Vellore, India; 24 Department of Neurosurgery, North West General Hospital and Research Center, Peshawar, Pakistan; 25 Department of Neurosurgery, Moscow State University of Medicine and Dentistry, Moscow, Russian Federation; 26 Department of Neurosurgery, Beijing Tiantan Medical Hospital, Capital Medical University, Beijing, China; 27 Department of Neurosurgery, Antwerp University Hospital and University of Antwerp, Antwerp, Belgium; 28 Department of Neurological Surgery, University of California San Francisco, San Francisco, CA USA; 29 Department of Plastic and Reconstructive Surgery, Johns Hopkins Hospital, Baltimore, MD USA; 30 Anesthesia and Intensive Care Unit, Department of Surgical Sciences, University of Torino, Torino, Italy; 31 Division of Anaesthesia, Addenbrooke’s Hospital and University of Cambridge, Cambridge, UK; 32 Oakland University William Beaumont School of Medicine and Michigan Head & Spine Institute, Auburn Hills, MI USA; 33 Departments of Neurology, Anesthesia/Critical Care & Surgery, University of Massachusetts Medical School, Worcester, MA USA; 34 Department of Neurosurgery, University of Pittsburgh Medical Center, Pittsburgh, PA USA; 35 Global Neurosurgery Initiative, Program in Global Surgery and Social Change, Department of Global Health and Social Medicine, Harvard Medical School, Boston, MA USA; 36 Department of Neurosurgery, Alfred Hospital, Melbourne, Australia; 37 Department of Surgery, Monash University, Melbourne, Australia; 38 George Washington University School of Medicine and Health Sciences, Washington, DC USA; 39 INUB/MEDITECH Research Group, El Bosque University, Bogotá, Colombia; 40 MEDITECH Foundation, Clinical Research, Cali, Colombia; 41 Department of Neurosurgery, Muhimbili Orthopedic-Neurosurgical Institute, Dar es Salaam, Tanzania; 42 Department of Physiopathology and Transplantation, Milan University, Milan, Italy; 43 Neuroscience Intensive Care Unit, Department of Anaesthesia and Critical Care, Fondazione IRCCS Cà Granda Ospedale Maggiore Policlinico, Milan, Italy; 44 Department of Neurological Surgery, Penn State University Milton S. Hershey Medical Center, Hershey, PA USA; 45 Department of Neurosurgery, The Royal London Hospital, London, UK; 46 Queen Mary University of London, London, UK; 47 Department of Neurosurgery, Hofstra North Shore-LIJ School of Medicine, Hempstead, NY USA; 48 Department of Neurosurgery, Virginia Commonwealth University, Richmond, VA USA; 49 Neurosurgery Division, Faculty of Medicine, University of Malaya, Kuala Lumpur, Malaysia; 50 Department of Neurosurgery, Royal Adelaide Hospital, University of Adelaide, Adelaide, South Australia Australia; 51 Imperial Neurotrauma Centre, Department of Surgery and Cancer, Imperial College, London, UK; 52 Department of Neurosurgery, Humanitas University and Research Hospital, Milan, Italy

**Keywords:** Neurosurgery, Neurotrauma, Decompression, Cranioplasty

## Abstract

**Background:**

Two randomised trials assessing the effectiveness of decompressive craniectomy (DC) following traumatic brain injury (TBI) were published in recent years: DECRA in 2011 and RESCUEicp in 2016. As the results have generated debate amongst clinicians and researchers working in the field of TBI worldwide, it was felt necessary to provide general guidance on the use of DC following TBI and identify areas of ongoing uncertainty via a consensus-based approach.

**Methods:**

The International Consensus Meeting on the Role of Decompressive Craniectomy in the Management of Traumatic Brain Injury took place in Cambridge, UK, on the 28th and 29th September 2017. The meeting was jointly organised by the World Federation of Neurosurgical Societies (WFNS), AO/Global Neuro and the NIHR Global Health Research Group on Neurotrauma. Discussions and voting were organised around six pre-specified themes: (1) primary DC for mass lesions, (2) secondary DC for intracranial hypertension, (3) peri-operative care, (4) surgical technique, (5) cranial reconstruction and (6) DC in low- and middle-income countries.

**Results:**

The invited participants discussed existing published evidence and proposed consensus statements. Statements required an agreement threshold of more than 70% by blinded voting for approval.

**Conclusions:**

In this manuscript, we present the final consensus-based recommendations. We have also identified areas of uncertainty, where further research is required, including the role of primary DC, the role of hinge craniotomy and the optimal timing and material for skull reconstruction.

## Introduction

Traumatic brain injury (TBI) is a major public health and socioeconomic problem around the world. According to the World Health Organisation (WHO), more than 5 million people die every year as a result of trauma, accounting for 9% of the world’s deaths. Trauma also results in millions of non-fatal injuries leading to life-long disability [90]. Of all the types of traumatic injuries, those to the brain are the most likely to result in death or permanent disability [102]. It is estimated that 69 million (95% CI 64–74 million) individuals worldwide suffer a TBI each year. However, the true burden of TBI and its sequelae appears to be underestimated owing to incomplete capture of data, especially in low- and middle-income countries (LMICs) [79]. Nevertheless, LMICs face almost three times more cases of TBI proportionally than high-income countries (HICs).

Multiple large cohort studies of TBI patients have demonstrated intracranial hypertension to be independently associated with a higher risk of death and poor outcome following TBI [3,4,31]. Consequently, management of brain swelling and elevated intracranial pressure (ICP) is a key component of acute TBI care [14]. A foundation of current protocols and guidelines in TBI is the treatment and prevention of pathological elevation in ICP, which can compromise cerebral perfusion and lead to further neurological compromise and life-threatening brain herniation. Decompressive craniectomy (DC) is a neurosurgical procedure that involves removal of a section of the skull (‘bone flap’) and opening of the underlying dura. From a physiological viewpoint, it provides additional space for the swollen brain to decompress, leading to reduction in ICP and maintained or improved cerebral compliance [9, 99]. Described more than a century ago by Theodor Kocher [47], the utility of the procedure and its effect on patient outcome were perpetually questioned, and it went in and out of fashion throughout the twentieth century. DC is a major operation associated with significant early and late complications including seizures, subdural hygroma, hydrocephalus, and infection. Furthermore, it necessitates an additional operation for cranial reconstruction, termed cranioplasty, which is also associated with significant morbidity. Until this decade, the dearth of high-quality outcome data from randomised controlled trials caused uncertainty about the efficacy of DC, and DC did not feature in the Third Edition of the Brain Trauma Foundation Guidelines for TBI Management in 2007 [10].

In the last 10 years, the TBI community has witnessed considerable research efforts, including several randomised trials, to address such aspects of DC as surgical technique, timing and indications in different patient populations. Two of the trials were international and addressed distinct clinical questions. DECRA [24] assessed the efficacy of bifrontotemporoparietal DC in adult TBI patients with diffuse brain injury (i.e. no mass lesion requiring surgical evacuation) in whom first-tier intensive care and neurosurgical therapies had not maintained ICP below accepted targets. RESCUEicp [37] assessed the efficacy of DC after first- and second-tier therapies had failed to control refractory and sustained intracranial hypertension in TBI patients. The results of both trials sparked further debate in the TBI community worldwide. There was a palpable need for a consensus meeting to provide general guidance on the use of DC following TBI and to identify areas of ongoing uncertainty.

The International Consensus Meeting on the Role of Decompressive Craniectomy in the Management of Traumatic Brain Injury was organised jointly by the World Federation of Neurosurgical Societies (WFNS), AO/Global Neuro and the NIHR Global Health Research Group on Neurotrauma and hosted at the University of Cambridge on the 28th and 29th September 2017.

## Methods

Delegates were invited to participate in the consensus meeting based on their experience and contributions to the literature on DC and/or TBI. The aim was to invite approximately 50 delegates with a broad geographic representation. The various topics of the consensus meeting were grouped into six themes to aid structured discussion and consensus building. The six themes were (i) primary DC for mass lesions, (ii) secondary DC for intracranial hypertension, (iii) peri-operative care of patients undergoing DC, (iv) surgical technique, (v) cranial reconstruction following DC and (vi) DC in LMICs.

The meeting took place over 2 days. On the first day, delegates worked in six small groups, coordinated by two facilitators, with the aim of discussing the relevant literature, identifying areas of agreement/disagreement and eventually generating approximately 10 relevant recommendations for clinical practice and areas of uncertainty for future research. On the second day, all delegates initially listened to the small group facilitators outlining key points from their small group’s discussion and presenting the proposed recommendations, elaborating as necessary. Each small group presentation was followed by floor discussion which provided the opportunity for all delegates to debate the recommendations proposed by the small groups and refine them if necessary prior to anonymous voting. Anonymous real-time voting of all proposed recommendations with the participation of all delegates took place with the use of audience engagement software (Glisser Limited, London, UK). When voting as to whether to include each proposed recommendation in the final consensus statement, the three options were ‘agree’, ‘uncertain’ and ‘disagree’. The threshold for including a recommendation in the final statement was a supermajority of 70% of delegates in agreement. Additionally, recommendations which during the first round of voting gathered levels of agreement close to 70% (i.e. just above or just below), high levels of uncertain responses or similar levels of agreement and disagreement were refined following floor discussion and put to a second round of voting. Following each statement, we present the percentage level of agreement followed by the ratio of participants voting in favour to the total number of participants casting their vote.

For the purpose of the consensus statement, we performed a literature search in PubMed using decompressive craniectomy and traumatic brain injury as key words. Bibliographies of retrieved reports were then searched for additional references. Only articles published in the English language were included.

## Results (including discussion and consensus statements)

### Primary DC for mass lesions

Primary DC refers to leaving a bone flap out after evacuating an intracranial mass lesion: an extradural, subdural and/or an intraparenchymal traumatic haematoma or contusion. The rationale is to control ICP in the post-operative period. The disadvantage is that later skull reconstruction is required. Traumatic haematomas are present in approximately 45% of severe TBI cases [11, 12, 13]. DC is a treatment option following evacuation of mass lesion at initial craniotomy, but the exact indications require further refinement.

Extradural/epidural haematomas (EDH) occur in approximately 2% of all head injuries and usually present as isolated lesions without significant intraparenchymal swelling. The Brain Trauma Foundation (BTF) guidelines on the management of EDH recommend craniotomy and evacuation for all patients with an EDH volume of greater than 30 ml regardless of Glasgow coma scale (GCS) score [11]. The Milan Consensus Conference on Clinical Applications of Intracranial Pressure Monitoring in Traumatic Brain Injury in 2014 reviewed evidence on ICP trends following evacuation of isolated EDH and found that there is low risk of intracranial hypertension developing [94], suggesting that DC is not routinely required for treatment of isolated EDH.

Acute subdural haematomas (ASDH) can be treated surgically with craniotomy (bone replaced) or DC (bone left out). They are present in approximately one third of severe TBI patients, but two thirds of TBI patients undergoing surgery (excluding external ventricular drain and ICP monitoring insertion) have an ASDH evacuated [22]. ASDHs are often associated with presence of intraparenchymal contusions or haematomas and with a propensity for brain swelling [22, 83, 88, 89]. BTF guidelines recommend immediate operative intervention if ASDH thickness is more than 10 mm or midline shift is greater than 5 mm regardless of the GCS score. They also recommend that patients with GCS scores less than 9, even if the thickness of ASDH is less than 10 or midline shift less than 8 mm, should undergo emergent surgical evacuation of the lesion if the GCS score decreases by 2 or more points from the time of injury to the time of hospital admission, or if the patient presents with asymmetric or fixed and dilated pupils [12]. BTF guidelines recommend that surgical evacuation should be performed using a craniotomy with or without replacement of the bone flap, but do not specify the exact indications for craniectomy. There are variations in clinical practice around the world when it comes to ASDH evacuation, with some neurosurgeons performing primary DC more readily and more frequently than others. Two international surveys of practices found primary DC to be performed most frequently because the brain is bulging beyond the inner table of the skull intraoperatively, preventing the safe replacement of the bone flap, or because there is concern that the brain may swell further in the post-operative period^,22,49^.

Early studies evaluating ICP following craniotomy for ASDH evacuation found that a significant proportion of patients develops intracranial hypertension postoperatively. A study by Miller and colleagues [62] showed that two-thirds of the 48 patients with an evacuated acute SDH had intracranial hypertension in the postoperative period (defined as persistently raised ICP above 20 mmHg), with just over half of those patients developing uncontrollable intracranial hypertension leading to herniation and death. In the series of Wilberger and colleagues [110], which examined 101 comatose patients who had a craniotomy for ASDH, 43% had sustained intracranial hypertension that was uncontrolled with standard therapy. The mortality in this group was 95% compared to 40% in the group of patients whose ICP remained under 20 mmHg. The theoretical advantage of DC would therefore be ICP reduction. However, it is not yet clear whether this necessarily translates into improved functional outcomes in all patient groups. To date, there is no published class 1 evidence. A number of retrospective observational studies and case series have compared the effectiveness of craniotomy and primary craniectomy in patients with ASDH, with conflicting conclusions. Some of the studies found worse outcomes in patients undergoing DC [16, 44, 56, 103, 112] whilst other studies did not reach the same conclusions [32, 33, 38, 58, 113]. These studies often had significant differences between the treatment groups, as patients who underwent primary DC tended to be the ones who had characteristics suggestive of more severe TBI: lower GCS score, thicker ASDH and greater midline shift as well as more significant extracranial injuries [32, 33, 38, 44, 58, 112, 113]. Thus, in the absence of a randomisation process, there is an obvious selection bias which makes generalisation of these findings difficult. More recently, a new research method has emerged, termed non-experimental clinical effectiveness research, which aims to utilise the heterogeneity in systems, practices and outcomes in order to compare the effectiveness of interventions that may be standard practice in some centres or systems, but not in others. One such study by Hartings and colleagues [33] compared practices and outcomes between two neurosurgical centres and found postoperative ICP to be better controlled and patient outcomes better in the centre with greater utilisation of primary DC. They also found that patients requiring evacuation of subdural hematomas and contusions may benefit from DC in conjunction with lesion evacuation, even when elevated ICP is not a factor in the decision to perform surgery [33].

The RESCUE-ASDH study is a multicentre, pragmatic, parallel group randomised trial which aims to compare the clinical and cost effectiveness of primary DC versus craniotomy for the management of adult head-injured patients undergoing evacuation of an ASDH [48, 76]. The trial includes adult head-injured patients (16 years of age and above) who have an ASDH on CT (patients with additional lesions such as intracerebral haemorrhage and contusions can be included) and the admitting neurosurgeon feels that the ASDH needs to be evacuated either by a craniotomy or DC with the bone flap of at least 11 cm in both instances. Exclusion criteria are bilateral ASDHs both requiring evacuation, previous enrolment in RESCUE-ASDH study or severe pre-existing physical or mental disability or severe comorbidity which would lead to a poor outcome even if the patient made a full recovery from the head injury. Eligible patients are randomised to craniotomy or DC intraoperatively after evacuating the ASDH. Patients with significant brain swelling preventing safe replacement of the bone flap are not suitable for randomisation and are being followed up in an observational cohort. The primary outcome measure is the extended Glasgow outcome scale (GOSE) at 12-month post-injury [48, 76]. Recruitment for the study will complete in 2019.

DC is associated with significant morbidity and necessitates an additional operation (cranioplasty) to reconstruct the cranium. An introduction of an alternative surgical method to DC, termed hinge craniotomy, has gained traction in the last 10 years. Hinge craniotomy is a surgical procedure whereby the bone flap is replaced before closure in such way that it is secured at one bone edge with a titanium plate attachment, and the other edges of the bone flap have a plate attachment secured only on the bone flap (but not connected to the cranial bone edge). This allows the bone flap to expand outward, but prevents it from sinking inward toward the brain [84]. Several retrospective studies are now available looking at mixed cohorts of DC patients (TBI and stroke) which demonstrate ICP control comparable to DC [41,46,84]. Additional good-quality studies are required to assess the effect on outcomes [50].

There are variations in clinical practice regarding use of continuous ICP monitoring following primary DC [49]. There is no class I evidence available, but retrospective observational studies looking specifically at ICP trends following DC support its use [70]. Picetti and colleagues [70] conducted a retrospective analysis of prospectively collected data on ICP, CPP and functional outcome in patients following primary DC. The authors have found that episodes of intracranial hypertension associated with low CPP occur frequently after primary DC. These were associated with unfavourable neurological outcome. They found that, despite DC, there was still a correlation between raised ICP and poor functional outcome. They conclude that ICP monitoring is clinically useful in guiding therapy after primary DC [70].

Penetrating brain injury (PBI) was briefly discussed during the consensus meeting. The meeting delegates acknowledged the pathophysiology of penetrating brain injury to be different from that of blunt injury. There are no clinical trials to date to assess the role of DC in PBI. Practice is based on case series and has been driven by recent extensive military experience. Brain swelling is often severe, and intracranial hypertension can be relieved by large DC. Generally, early wide DC seems to be in use for severe TBI with diffuse pathology [6, 8, 26, 68, 74, 78].

#### Consensus statements concerning primary DC for mass lesions

1. After evacuating an acute subdural haematoma (ASDH), if the brain is bulging beyond the inner table of the skull intra-operatively, consider leaving the bone flap out based upon clinical and radiographic findings (100%; *49/49)*
2. After evacuating an ASDH, if the brain is very relaxed and the pre-operative computed tomography (CT) imaging is not in keeping with a high risk of progressive brain swelling (i.e. no or minimal parenchymal injury), the bone flap should be replaced (e.g. elderly patient, low-energy mechanism). (87.8%; *43/49*)
3. For the intermediate category of ASDH patients (brain neither very relaxed, nor bulging), surgeon judgement must be used to decide whether to leave the bone flap out or not. (97.9%; *47/48*)
4. After evacuating an isolated epidural haematoma, the bone flap, in general, should be replaced (87.8%; 43/49)
5. In a primary DC, the bone flap should be of large size, at least 12 cm × 15 cm (93.9%; 46/49)
6. DC is an option for patient with contusions in whom contusions are not being evacuated (83.3%; 40/48)
7. If well-circumscribed contusions/intraparenchymal haematoma are present, surgeon judgement should be used to decide whether to evacuate the contusions/intraparenchymal haematomas (95.8%; 46/48)
8. When the bone is replaced, an ICP monitor should be placed where available (74.5%; 35/47)
9. An ICP monitor should be placed following primary DC, if available (77.1%; 37/48)
10. In situations where no invasive/continuous ICP monitoring is available, computed tomography (CT) imaging should be used to monitor progress (97.9%; 47/48)

#### Areas of uncertainty regarding primary DC for mass lesions

1. For intermediate category of ASDH patient, where following evacuation of ASDH, brain is neither relaxed nor bulging, it is not clear if performing DC instead of replacing a bone flap provides any additional benefits for the patient. The results of the RESCUE-ASDH trial are awaited.
2. The use of hinge craniotomy requires further evaluation to determine effects on outcome.

### Secondary DC

A secondary DC is typically used as part of tiered therapeutic protocols that are frequently used in intensive care units (ICUs) in order to control raised ICP after TBI. A secondary DC can be undertaken as last-tier life-saving therapy for patients with refractory intracranial hypertension (i.e. when all other measures have failed to reduce ICP) or as a second-tier therapy in patients with less pronounced elevation of ICP (i.e. as a neuro-protective measure).

Studies in patients undergoing secondary DC found it to be effective in reducing ICP and improving CPP [1,19,24,29,34,37,52,64,66,67,82,85,100,108], reducing the cumulative ischaemic burden and therapy intensity level [106]. The effect of secondary DC on functional outcomes is not as straightforward. Many observational studies and case series attempted to address the question, with wide variation in reported outcomes. This is not surprising given that they include very heterogeneous populations and measure outcomes at different time points using different outcome measures. There are only a handful of international (multicentre), randomised trials.

#### Randomised controlled trials on secondary DC

The first randomised controlled trial (RCT) of early secondary DC for TBI was performed in paediatric patients [97]. Twenty-seven children (median age, 120.9 months; range, 13.6–176.4 months) with head injuries were randomly assigned to receive medical management alone or medical management plus bitemporal DC (removal of a disc of temporal bone measuring about 3–4 cm, with extension of the craniectomy to the floor of the middle cranial fossa; dura mater was not open). The study showed that DC patients are more likely to have better ICP control with lower ICP values following DC and fewer episodes of intracranial hypertension, and functional outcome and quality of life may be better than in children treated with medical management alone (*P* = 0.046; owing to multiple significance testing *P* < 0.0221 is required for statistical significance).

Results of the Decompressive Craniectomy in Diffuse Brain Injury (DECRA) international, multicentre RCT were published in 2011 [24]. Between December 2002 and April 2010, the trial collaborators randomly assigned 155 adults with severe diffuse TBI to either bifrontotemporoparietal DC or standard (medical) treatment if they developed intracranial hypertension defined as ICP of more than 20 mmHg for more than 15 min in a 1-h period refractory to first-tier therapies [24]. The original primary outcome was an unfavourable outcome (a composite of death, vegetative state, or severe disability), as evaluated on the Extended Glasgow Outcome Scale 6 months after injury. Patients in the DC group had less time with ICPs above the treatment threshold (*p* < 0.001), fewer interventions for increased ICP (*p* < 0.02 for all comparisons), and fewer days in the ICU (*p* < 0.001). However, better ICP control did not translate into improved outcomes for the DC patients. Mortality was similar in the two treatment groups (19% in DC group and 18% in control group), but unfavourable outcome (composite of death, vegetative state or severe disability, GOS-E 1–4) was higher in the DC group compared to the control group (70% vs 51%; odds ratio, 2.21; *p* = 0.02). While the two groups were well-matched for most variables, there was a higher proportion of patients with bilateral unreactive pupils in the DC group (27% vs 12% in the medical treatment group; *p* = 0.04). Following post hoc adjustment for baseline pupil reactivity, there was no difference in the rates of unfavourable outcomes between the two treatment groups [24].

Randomised Evaluation of Surgery with Craniectomy for Uncontrollable Elevation of Intracranial Pressure (RESCUEicp), an international, multicenter, parallel-group, superiority RCT, compared last-tier secondary DC with continued medical management for refractory intracranial hypertension after TBI [37]. The trial included patients with TBI aged between 10 and 65 years, with an abnormal computed tomography scan of the brain, an ICP monitor in place and raised ICP of more than 25 mmHg for 1 to 12 h, despite applying stage 1 and 2 measures of the ICP protocol (for details on protocol, see reference 37). The surgical treatment was either large unilateral frontotemporo-arietal craniectomy (hemicraniectomy) or bifrontal craniectomy. The primary outcome measure was the Extended Glasgow Outcome Scale (GOSE) at 6 months after randomisation. Four hundred and eight patients were recruited to this trial: 206 were randomised to the surgical and 202 to the medical group. No significant between-group variability was observed in terms of baseline characteristics and medical or surgical treatment given prior to randomisation. At 6 months, the DC group had a significantly lower mortality rate compared to the medical group (26.9 vs 49.9%). Surgery was found to be associated with higher rates of vegetative state, lower severe disability and upper severe disability than medical management, but the rates of moderate disability and good recovery were comparable between the groups [37]. The trial pre-specified favourable outcome as upper severe disability or better on the GOSE and with such a pre-specified sensitivity analysis, 42.8% of surgical patients had a favourable outcome compared to 34.6% of medical patients (*p* = 0.12). The same pre-specified sensitivity analysis at 12 months showed that 45.4% of surgical patients had a favourable outcome compared to 32.4% of medical patients (*p* = 0.01). The trial also showed that control of ICP was better in the surgical than the medical group.

The 4th Edition of the BTF guideline [14] does not recommend ‘bifrontal DC to improve outcomes as measured by the GOSE score at 6 months post-injury in severe TBI patients with diffuse injury (without mass lesions), and with ICP elevation to values > 20 mm Hg for more than 15 min within a 1-h period that are refractory to first-tier therapies’. This recommendation is based on level IIa evidence from a single class 1 study [DECRA [24]]. The guidelines also acknowledge that DC is a new topic in the 4th Edition of the BTF Guidelines for the Management of Severe TBI and that as they were published before the publication of the RESCUEicp trial, the trial results will be included in a future guideline update [14].

#### Consensus statements concerning secondary DC

1. Where available, ICP monitoring is needed as a component of decision making for secondary DC (95.7%; 45/47)
2. Secondary DC is effective in decreasing ICP, but underlying brain pathology and pathophysiology contribute to overall outcome (95.8%; 46/48)
3. The optimal candidate for secondary DC is a patient whose ICP elevation is the primary contributor to poor outcome and the primary injury is deemed compatible with acceptable recovery (97.9%; 46/47)
4. Where available, ICP monitoring should be used in conjunction with CT findings and neurological exam to decide on secondary DC (100%; 47/47)
5. Use of ICP monitoring is recommended according to regional availability of resources; non-invasive monitoring methods to establish refractory intracranial hypertension can be used (79.2%; 38/48)
6. While secondary DC is a potentially useful operation, it should be applied selectively as there is uncertainty as to which severe TBI subgroups will truly benefit (87.5%; 42/48)
7. DC may decrease mortality. However, it is not benign and is associated with significant risks of complications and potentially increased risks of disability (93.8%; 45/48)
8. Before contemplating secondary DC, providers should conduct frank discussions with family members/surrogates regarding the risks, benefits, alternatives and potential prognosis (97.9%; 46/47)
9. Simple and standard ICP thresholds alone are not sufficient to determine eligibility for secondary DC. Sustained, refractory ICP elevations in conjunction with other clinical parameters (e.g. examination, imaging, non-invasive technologies, other monitoring modalities, status of underlying brain physiology) should be considered when making the decision to perform secondary DC (95.7%; 45/47)
10. ICP monitoring should continue after secondary DC. Thresholds for intervention after decompression may be reconsidered (76.6%; 36/47)
11. We recommend a large DC with opening of the dura to effectively reduce ICP and reduce incidence of secondary cortical injury from reduced venous drainage (91.5%; 43/47)
12. Bifrontal or unilateral DC are options in the surgical treatment of diffuse TBI (95.6%; 44/46)

### Peri-operative care

The BTF guidelines (4th Edition) recommend management of severe TBI patients using information from ICP monitoring to reduce in-hospital and 2-week post-injury mortality [7]. The accepted threshold for treatment is 20 mmHg [14, 94]. Further, it is the burden of intracranial hypertension (duration as well as severity) that is related to poor outcomes [14, 31, 40, 94].

Chesnut and colleagues [17] conducted a multicentre randomised controlled trial that compared outcomes for patients whose treatment was informed by continuous (invasive) ICP monitoring with those whose treatment was informed by serial imaging (157 patients) and clinical examination (167 patients). This high-quality study found that 6-month outcomes for patients managed with information from clinical assessment do not differ from those for patients managed with information from the ICP monitor [17]. Patients in both arms received protocolised tiered ICP-lowering therapies, and 30% of patients in each arm received a DC. It is important to emphasise that this trial does not challenge the fundamental concept that brain oedema and raised ICP should be actively managed in patients with TBI (with medical and/or surgical means).

#### Consensus statements concerning peri-operative care

1. Think of ICP as a dose—hours for ICP 25–30 mmHg, minutes for ICP 30–40 mmHg (78.4%; 40/51)
2. Full escalation of treatment should be achieved before DC, unless clinical deterioration prompts more urgent surgery (93.6%; 44/47)
3. Follow usual local protocols for major cranial surgery with regards to haemostasis and antibiotics (97.9%; 46/47)
4. Maintain ICP therapy during surgery and immediately post-DC unless limiting side effects (86.3%; 44/51)
5. If ICP is well controlled by DC:
  the treatment bundle for intracranial hypertension should not be immediately terminated or altered after DC (82.4%; 42/51)
  a postoperative CT within 24 h of surgery is recommended to document DC effectiveness and document complications (80.4%; 41/51)
6. Continue ICP monitoring and ICP therapy till ICP known to be controlled and stable; de-escalation should be staged with sodium normalisation last (80.4%; 41/51)
7. If ICP is not controlled by DC:
  Continue therapy if there is no change in salvageability as assessed by pupils, clinical state and CT head imaging (91.5%; 43/47)
  Look for reversible reasons for intracranial hypertension (electroencephalogram, CSF circulation disorders) (96.1%; 49/51)
  Consider brief sedation hold to assess neurologic exam, accepting mild intracranial hypertension (78.7%; 37/47)
8. If uncontrollable intracranial hypertension occurs following DC, re-assess the situation and treatment goals (98%; 50/51)

### Surgical technique in DC

There are three main approaches to secondary DC: bifrontal craniectomy, unilateral frontotemporoparietal craniectomy (hemicraniectomy) and bilateral hemicraniectomy. Once the bone is removed, the dura is opened to relieve intracranial hypertension.

Reithmeier and colleagues [75], in a study in five patients undergoing DC, elegantly showed reduction in ICP and increase in brain tissue oxygen tension (PbtO2) and CPP with every step of DC: removal of bone flap, opening of the dura and skin closure. After removing the bone flap, ICP values dropped to physiological values (mean: 7.4 mmHg), whereas PbtO2 values increased only slightly (mean: 11 mmHg). Opening of the dura resulted in a further decrease of ICP (mean 4.8 mmHg) and an increase of PbtO2 to normal limits (mean: 18.8 mmHg). After skin closure, mean ICP was 6.8 mmHg and mean ptiO2 was 21.7 mmHg, respectively. They found a significant decrease of ICP after craniectomy (*p* < 0.042) and after dura enlargement (*p* < 0.039) as well as a statistically significant increase in PbtO2 after craniectomy (*p* < 0.043) and after dura enlargement (*p* < 0.041). The results suggest that dura enlargement is the crucial step to restore adequate brain tissue oxygenation [75].

Two RCTs, one multicentre [39] and one single-centre [73], evaluated the effects of craniectomy size on functional outcomes. Both studies compared unilateral frontotemporoparietal craniectomy (STC) with a bone flap size of 12 × 15 cm to a limited smaller (LC) temporoparietal craniectomy (8 × 6 cm). Jiang and colleagues [39] found STC to be associated with lower mortality (26.2%) compared to LC (35.1%; *p* < 0.05). GOS of 4–5 was reached by 39.8% of STC patients compared to 28.6% of LC patients (*p* = 0.05). Further, the incidence of delayed haematoma and incisional CSF fistula significantly was lower in the STC group, while other complications did not differ [39]. Qiu and colleagues [73] found the STC to be associated with a larger reduction of ICP. Mortality rates at 1 month after treatment were 27% in the SLC and 57% in the LC group (*p* = 0.010). Good neurological outcome (GOS score of 4 to 5) rates 1 year after injury for the groups were 56.8% and 32.4%, respectively (*p* = 0.035). The incidences of delayed intracranial haematoma and subdural effusion were 21.6% and 10.8% versus 5.4% and 0, respectively (*p* = 0.041 and 0.040) [73].

The topic of hinge craniotomy has already been discussed in the section on primary DC for mass lesions and will not be repeated here.

#### Consensus statement concerning surgical technique in DC

1. Skin incision should be larger than the intended craniotomy and pinna should be avoided (92%; 46/50)
2. Bone flap size should be large and incorporate removal of bone to the middle cranial fossa floor for both unilateral and bifrontal decompressive craniectomies (94%; 47/50)
3. The dura should be opened and primary dural closure should not be performed (98%; 46/47)
4. Expansile dural graft should be used—sutured duraplasty or onlay (91.5%; 43/47)
5. Avoidance of techniques that will contribute to secondary injury is important, e.g. galeal adhesion to cerebral tissue, migration of dural graft material, excessive graft or hemostatic material volumes (86%; 43/50)

#### Areas of disagreement or uncertainty regarding surgical technique in DC

1. The optimal materials for duraplasty
2. The necessity of sutured expansile duraplasty
3. The role of lobectomy/contusion removal
4. The method of bone flap storage
5. The removal of bone overlying the superior sagittal sinus when performing bifrontal DC and superior sagittal sinus division
6. The role of hinge craniotomy

### Cranial reconstruction following DC (cranioplasty)

Cranial reconstruction (cranioplasty) following DC restores the original skull contour. Large skull defects following trauma DC leave the brain unprotected and hinder ICP regulation, CSF dynamics and cerebral blood flow, potentially giving rise to complications such as hydrocephalus and the syndrome of the trephined [54,69].

The importance of cranial reconstruction extends beyond cosmesis. Cranioplasty offers brain protection and restores the integrity of the calvarial vault and thus can restore normal cerebrospinal fluid dynamics [53,54,111]. CT perfusion [107] and transcranial Doppler ultrasonography [92] have shown improvement in cerebral blood flow following cranioplasty. There are now multiple reports, some specifically from TBI patient cohorts [7,20,23,35,36,43,53,60,61,68,116], suggesting that cranioplasty aids neurological recovery. Skull contour restoration is of particular importance to patients cosmetically and may improve their psychosocial interactions and quality of life in general. It remains unclear how timing of cranioplasty affects neurological recovery as there is clear lack of class 1 evidence.

Cranioplasty operations carry a significant risk of postoperative complications, which can develop in the immediate postoperative course or months and years later. The overall rate of complications has been reported as 10.9–40.4% [2,5,15,18,21,25,27,45,51,55,57,59,63,71,77,80,86,93,101,104,105,109,115]. Autologous bone resorption may develop if the devitalised bone flap does not come into contact with the vascular bone edge and has been reported to occur with a frequency of 0.7–17.7% [45,55,71,104,105]. Surgical site infections are another concerning complication of cranioplasties and are reported in 5–12.8% of cases [45,55,71,95,104,105].

The optimal timing for cranioplasty remains unclear. Traditionally, surgeons would wait several months following DC to allow for recovery from the initial neurological insult and to ensure that cerebral oedema and inflammation have subsided. Multiple observational studies of the timing of cranioplasty have failed to detect a strong correlation between the timing and complications rates [2,5,15,18,21,25,27,45,51,55,57,59,63,71,77,80,86,93,95,101,104,105,109,115], and high-quality randomised trial data are lacking.

The ideal material used for cranioplasty should be lightweight, durable, malleable and easily fixable to the skull. A number of materials have been used in cranial reconstruction, from autologous bone flap (either implanted in the anterior abdominal wall or cryopreserved) to synthetic materials (methyl methacrylate, polyethylene, ceramic, glass or titanium plates). Plates can be made manually or, more recently, by using computer-based additive manufacturing technology to produce customised patient-specific implants. Despite the large number of studies in the literature [30,42,72,87,96,98], solid data comparing the various materials with regards to clinical outcome, patient satisfaction, complication rates and costs are lacking.

#### Consensus statements concerning cranial reconstruction following DC

1. Cranioplasty is required for limited brain protection and reconstruction of the cranial contour. It can possibly improve neurological outcome (88.2%; 45/51)
2. Patients should be surgically and medically optimised before performing cranioplasty (98%, 50/51)
3. Optimal timing for cranioplasty is not clear (82.4%; 42/51)
4. Early cranioplasty may be safe and beneficial and requires further study (90.2%; 46/51)
5. There is no consensus on optimal material (96.1%; 49/51)
6. Cranioplasties are associated with significant complications and meticulous care (equal to other operations involving implants) should be exercised. Cranioplasties should be performed by competent surgeons (100%; 51/51)

#### Areas of disagreement or uncertainty regarding cranial reconstruction following DC

1. The optimal timing of cranioplasty (i.e. early vs late) remains unclear, with high-quality multi-centre studies lacking.
2. The optimal material for cranioplasty remains unclear, with high-quality multi-centre studies lacking.
3. A recent systematic review suggests that early cranioplasty (within 3 months) is associated with greater neurological improvement. This finding needs to be corroborated by randomised trials.

### DC in low- and middle-income countries

Trauma accounts for 9% of the world’s deaths. Approximately 90% of these injury-related deaths occur in low-income and middle-income countries (LMICs). Injuries to the brain and spine also result in millions of non-fatal injuries and consequent disability. LMICs are experiencing the greatest rate of increase in these injuries, largely because of road traffic accidents. Incomplete collection of epidemiological data makes quantification of the true worldwide magnitude and burden of trauma and TBI very difficult, and the data presented above may be underestimated. It is undeniable that TBI has significant clinical, economic and societal implications [28,65,79,81,91,114].

TBI care, including prevention, prehospital care, specialised neurotrauma care and rehabilitation, is complex and costly. In resource-constrained settings with limited or inadequate infrastructure and professional capacity and with fragile health systems with non-existent or poor social support, TBI treatment is even more challenging, and the application of current treatment protocols and guidelines, developed largely in high-income countries, may not be readily applicable [91]. Treatment paradigms need to be contextualised for the relevant health system, taking into account societal, cultural and ethical considerations and acceptable outcome measures.

Intensive care, ICP monitors and facilities for utilising hypothermia and barbiturate coma are not readily available in many resource-poor settings. Neurosurgeons may be more likely to use DC as a means of ICP control [81,91].

#### Consensus statement concerning DC in LMICs

1. The results of DECRA and RESCUEicp are not generalizable to conditions in LMICs and as such cannot change clinical practice in these areas (84.3%; 43/51)
2. We recognise and support that decisions about DC must be made in context of local knowledge of access to medical resources, capacity for long term care and cultural beliefs (98%; 50/51)
3. We recognise that most DCs in LMICs are primary DCs and support that due to limited resources valid indications for DC may be made by the clinical condition of the patient and the initial or most recent CT scan findings (90.2%; 46/51)
4. The decision to perform a DC should be made by the attending neurosurgeon responsible for the case (90.2%; 46/51)
5. DC is an invasive procedure with substantial potential for harm and should be done by a neurosurgeon or a neurosurgical trainee who is adequately trained for the procedure. Where neurosurgeons are not available, in exceptional circumstances, adequately trained surgeons may perform the procedure (87.2%; 41/47)
6. Regional authorities should be encouraged to promote availability of neurosurgeons to care for brain-injured patients (100%; 47/47)

## Conclusions

DC has been in and out of vogue for many decades. Against a background of a number of observational studies, two randomised trials assessing the effectiveness of DC (DC) following TBI have now been published and yield class 1 evidence. However, because there remain areas of uncertainty, the first International Consensus Meeting on the Role of DC in the Management of Traumatic Brain Injury was convened. The consensus conference addressed six pre-specified themes: (1) primary DC for mass lesions, (2) secondary DC for intracranial hypertension, (3) perioperative care, (4) surgical technique, (5) cranial reconstruction and (6) DC in LMICs. We have provided consensus statements to aid decision-making and also flagged areas of disagreement or uncertainty which require further investigation. In particular, the vast majority of studies have been conducted in high-income countries even though 89% of head injuries occur in LMICs. Hence, caution is required in applying evidence to management across the globe. Further research is required in several areas, including primary DC, the role of hinge craniotomy and the optimal timing and material for skull reconstruction.
